# Designing and prototyping of a reconfigurable segmented fan concrete shell as a flooring system

**DOI:** 10.1007/s44150-025-00126-6

**Published:** 2025-01-15

**Authors:** Mishael Nuh, Robin Oval, John Orr

**Affiliations:** 1 Department of Engineering, University of Cambridge, Cambridge, UK; 2 Faculty Of Civil Engineering and Geosciences, Delft University of Technology, Delft, Netherlands

**Keywords:** Circular construction, Reconfigurable systems, Sustainability, Concrete structures, Digital fabrication, Form-finding

## Abstract

A significant portion of the environmental impact of a building’s superstructure lies in its structural flooring. By leveraging funicular forms such as thin concrete shells, a materially and carbon-efficient alternative to bending-active flooring systems can be attained. In addition, through segmentation and the use of dry jointed interfaces, a segmented concrete shell allows for ease of disassembly compatible with circular economy principles for the built environment. This paper presents a novel segmented concrete shell flooring system that leverages the symmetry of revolution of the classical fan vault form to facilitate future design flexibility through increased reconfigurability. The design and form-finding of the segmented fan concrete shell are detailed through the use of an evolutionary algorithm and finite element analysis. Quarter-scale prototypes were digitally fabricated using a robotic concrete spraying process which were then assembled and tested to assess its structural potential, evaluate the limitations, and identify areas of future work. An embodied carbon analysis demonstrates that the system provides a mass and embodied carbon saving compared to conventional flooring systems while adding approximately a 20% embodied carbon premium over a comparable non-reconfigurable segmented shell flooring system. Rephrased, the proposed system provides a positive embodied carbon saving if enabling design flexibility through reconfiguration increases the life-span of the system by at least 20%. Through this work, it is shown that a segmented fan concrete shell presents a viable lightweight and carbon-efficient flooring system which has the potential to become a sustainable alternative that enables disassembly, reuse, and even reconfigurability for circular construction provided further research and development to address its current limitations for adoption in industry practices.

## Introduction

### Thin-shell vaulted concrete and masonry structures

Vaulted structures such as arches and shells present a materially efficient means of spanning large distances using low/no tensile capacity materials such as concrete and masonry. This is achieved by resisting loads through membrane action as opposed to bending; tensile stresses which can lead to cracking and brittle failure can be avoided, and internal tensile reinforcement can be altogether excluded. Such structures have been prevalent historically 1) when it was the only means of spanning large distances before the advent of steel reinforcement and pre-tensioning for masonry and concrete (e.g., cathedral vaults, masonry arches, etc.), 2) where stone was more widely available compared to wood, or 3) when labour costs were low compared to material costs and material efficiency often results in cost savings (e.g., thin concrete shell forms of Nervi [1] and Candela [2]). However, as labour costs increased, the extensive falsework and formwork required to build and assemble such structures became cost-prohibitive to fabricate, and the simple but inefficient prismatic forms–such as rectangular beams and flat slabs–became more preferred.

Recently, there has been a growing interest in re-evaluating vaulted funicular forms as a sustainable construction practice by minimizing the material usage and embodied carbon of a structure [3–6]. This is in part driven by new digital fabrication techniques which facilitate the construction and assembly of these structures at an affordable cost. Building floors are of particular interest for this as they comprise a large part of the material and embodied carbon of a typical building’s superstructure [7, 8]. It should be noted that the use of thin-shell forms for roofs and flooring systems is not new; there are various precedence for it, a prominent set being the timbrel masonry vaults built by the Guastavino Fireproof Construction Company used in over 1000 buildings primarily in the United States with one of its advantageous being its fire resistance [9]. One of the recent digital fabrication approaches is to print or fabricate the formwork and subsequent formers (often using clay, foam, or plastic) which is then cast with concrete [4, 10, 11]. A different approach is to use digital fabrication to extrude and place the material itself such as through sand 3D printing [5], extrusion-based printing [6], and also robotic concrete spraying [12, 13]. Regardless of the method, the goal is to employ robotically assisted techniques in order to reduce the labour costs associated with the fabrication of curved thin shell forms. The compression-only behaviour of funicular forms makes them suitable for disassembly and reuse, allowed by simple reversible joints, in order to aid in the formation of a circular economy for the built environment. However, these forms are generally bespoke, lacking repetition and modularity, and therefore present challenges for reconfigurability.

Further challenges are present with the construction of funicular systems in regards to their assembly. Traditional masonry requires the erection of a support structure which is often as geometrically complex as the final structure itself. This results in two structures being constructed: a support structure (which is later disassembled) and the final shell. In order to enable funicular structures to penetrate the commercial market and to be adopted by industry practices, this issue must be addressed to avoid extraneous labour costs.

### Circularity and reuse of concrete structures

Structural reuse presents challenges both to the design and construction aspects of a building project. In the prior, the design of structures needs to be governed by the existing stock of members, thereby presenting a reversal in thinking which traditionally details new component member sizes and specifications based on the design and analysis performed (also termed as a *form follows availability* design process [14]). As most designs are not formulated with the intent for disassembly, reuse, and reconfiguration, this presents difficulties for designers who wish to employ these pre-existing members. For example, spans of the new structure will then be dictated by the spans (and designed loading) of the available stock. Allowing for a degree of flexibility within the form of new builds with the intent of facilitating future builds will help ease this constraint. The advantages concerning sustainability are clear in that reusing structural components reduces both material usage, and retains the energy and embodied carbon already put in to manufacture the original components. The extended lifespan of these components is limited only by durability concerns; when well protected and in a safe environment, there are no technical limitations that prevent components from being continuously reused. It should be noted, however, that liability concerns (whether arising from actual durability concerns or merely from its perception) present additional barriers and may require a novel case-by-case approach to discussed between project parties and insurance companies [15, 16].

For concrete, the material poses further challenges compared to other conventional building materials such as steel and timber due to the lack of mechanical fasteners. Cast-in-place concrete results in monolithic interfaces and pre-cast members typically rely on grout and mortar to connect different components, all of which result in monolithic structures which are not easily dismantled. Despite these challenges which have posed hurdles to some concrete reuse projects in the past [17–20], there is great potential in concrete reuse in terms of cost and embodied carbon savings, with many of the hurdles identified as largely transitional [16]. Developing and employing structural systems that are designed with disassembly and reuse in mind will help to ease this transition and make the construction method more appealing for designers.

Funicular forms that employ membrane and arch action have the potential to alleviate these issues, both for the reuse of existing structures and for designs that facilitate reuse and circularity in the future. By avoiding the need for internal reinforcements, interruption of the pre-existing tensile reinforcement which may be necessary when cutting concrete members becomes non-problematic. This was demonstrated to great effect in the Re:Crete bridge which used concrete blocks sawn from cast-in-place concrete members to create an arch footbridge, using internal post-tensioning within and a tie to resolve the external thrust [21]. For designs enabling future reuse, funicular forms have the potential to minimize grouting and mortar use as segments do not need to have mortar in order to maintain their form and stability as the interfaces are held together through compressive forces – the simplest way to transmit forces. This was highlighted as a possible advantage for various segmented funicular structure prototypes ranging from bridges [6] to segmented funicular floors [5, 12]. In addition, the thrust is often resolved through the use of external ties which can also be post-tensioned. Because these tension members are external (as opposed to being embedded within concrete flat slabs and beams for bending), it makes disassembly much simpler. However, funicular structures pose challenges for reconfiguration in a reuse case with a scenario different from the previous one in terms of spans, supports, and loads; the segmentation and geometry of the forms are not conducive for spanning different distances compared to what they were initially designed and form-found for. While not a prerequisite for reuse, enabling designers to have extra flexibility in their design will help to lower the barrier to adopting component reuse with reconfiguration. Additionally, the form-found geometry of funicular structures often lends itself to multiple unique geometries for its segments which presents challenges for manufacturability at larger scales as well as for reuse (a lower number of unique segments means that replacements for damaged segments can be more easily sourced). As such, there is space to investigate whether a lightweight funicular structure can be designed such that it enables 1) disassembly, 2) reuse, and 3) reconfiguration for different spans.

### Problem statement and research objectives

Segmented thin-shell concrete floors present a structural system that has potentially large benefits for sustainability–previous works have demonstrated that vaulted thin-shell concrete structures can be a materially and carbon-efficient structural form [3–5]. However, as their segmentation and form do not have reconfiguration or component reuse as their primary objective, reuse of these shells dictates that the span of the shell be maintained, restricting designers and disincentivizing component reuse. The work detailed in this paper aims to leverage the structural form to create a design that also facilitates reuse through ease of disassembly and also by affording some design flexibility in the future through reconfiguration. All of this will be driven through recent digital fabrication advances to reduce the traditionally labour-intensive fabrication process for thin-shell concrete structures. Finally, translating the digital and engineering design work into the physical prototyping stage combined with structural assessment is necessary in order to demonstrate the viability of the flooring system and elucidate any shortcomings that may arise from manufacturing and physical constraints. In particular, the complexity of the assembly process compared to more developed and well-adopted systems, such as precast slab construction, are assessed through assembly of the prototype system. However, as the primary focus of the work is on the manufacturability and structural performance of the shell; assembly considerations, while acknowledged to be of great importance, are only assessed and left as future work.

### Contribution

This paper details the concept, design, fabrication, and prototyping of segmented fan concrete shells. The work demonstrates the potential of leveraging funicular forms for building systems compatible with circular economy principles and adds to the growing body of test data for segmented concrete structures. In Section “Design”, the design of the structure is detailed, along with the form-finding and optimization work performed to arrive at the final structure which is designed for disassembly and reconfigurability. Details regarding the fabrication of two quarter-scale prototypes measuring 2 m by 2 m using automated robotic concrete spraying are provided in Section “Fabrication and assembly of scale prototypes”. These prototypes were then tested under asymmetrical point loads until failure (Section “Structural load testing”), with the test modelled using nonlinear finite element analysis in Section “Structural numerical analysis”. A sustainability assessment is detailed in Section “Embodied carbon comparison”. Lastly, conclusions and future work is outlined in Section “Conclusions and future work”, highlighting areas of further development required to address current limitations, stressing the barriers towards industry implementation.

**Fig. 1:**
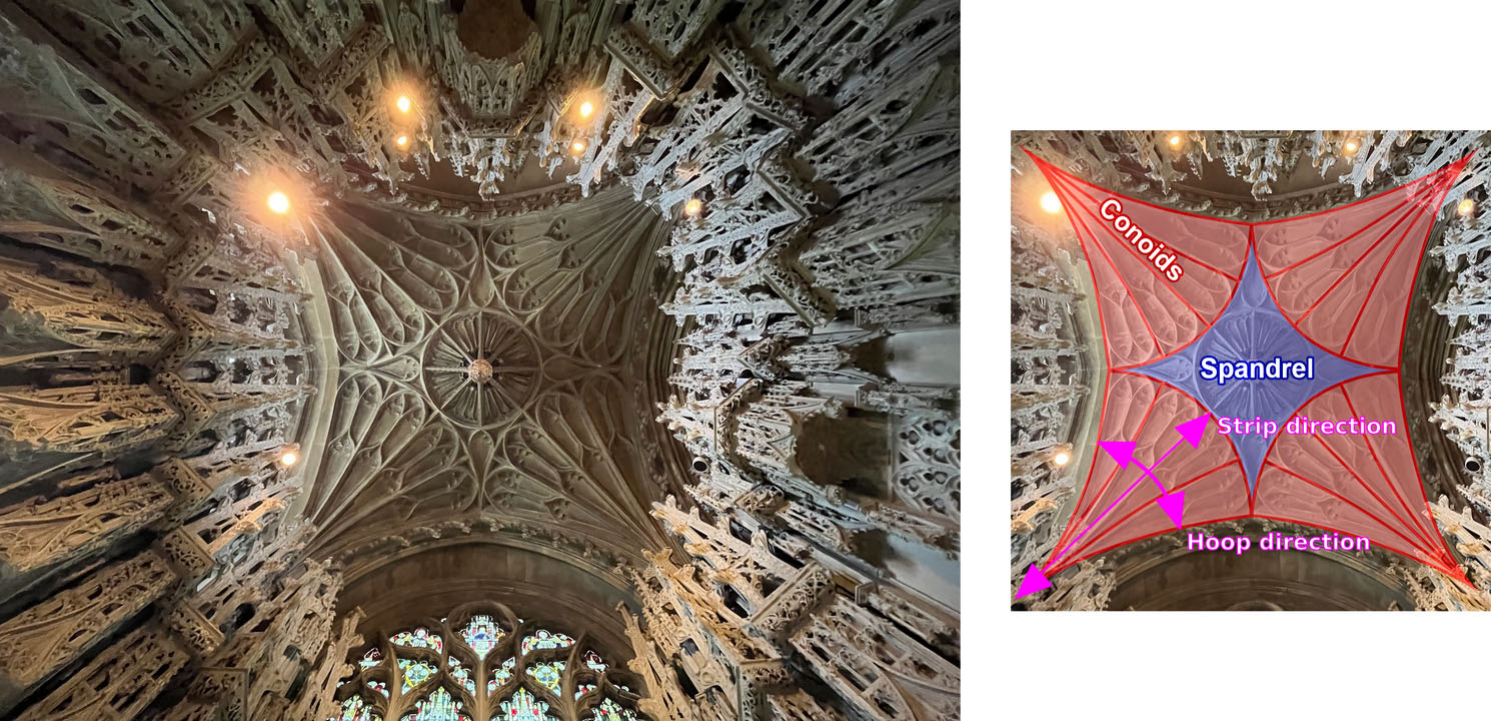
View of a fan vault in Ely cathedral with various parts highlighted (right)

## Design

### Fan vaults and segmentation

#### Form and geometry

The fan vault presents a unique shell geometry due to its local radial symmetry at each conoid which offers some particular advantages for standardisation and modularisation of the segmentation and prefabrication. The geometry itself can be divided into two distinct parts: the conoids which are created by revolving a generating curve around the column axis and the spandrel which fills the space in between the conoids (shown overlaid over a photo of a fan vault in Ely cathedral in Fig. 1). There are a variety of classifications of fan vault geometries based on how the conoids intersect [22]. For this work, it will be limited to the geometry where the conoids only intersect each other at points (i.e., no intersections which will cause the conoid geometry to be truncated) with a flat spandrel used to span the distance in between. This is in order to avoid truncating conoid segments and to maintain a high degree of repeatability between segments to ease fabrication (lower number of unique moulds and geometries to fabricate) and reuse (damaged segments can be more easily replaced).

The use of the flat spandrel to span the curved conoids takes the thrust surface outside of the geometry, thereby requiring some bending capacity within the spandrel. However, due to the thrust generated by the conoids, compressive stresses results in a pre-stressing effect on the concrete member, reducing the requirements for tensile reinforcement to properly resist the bending forces.

#### Segmentation

**Fig. 2:**
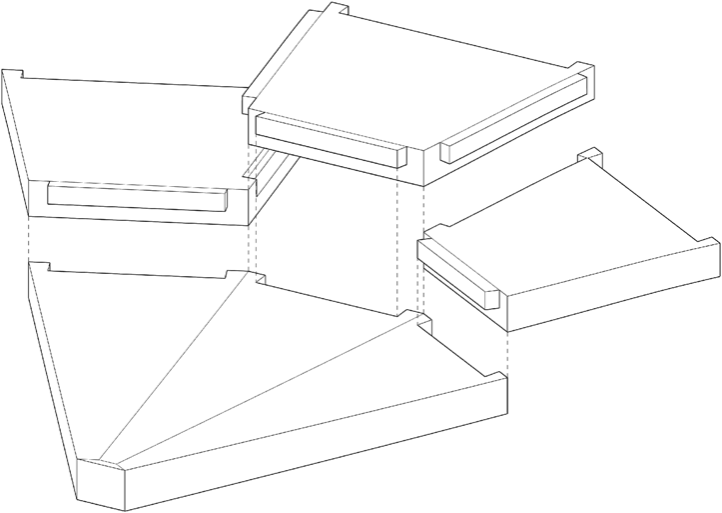
Shear keys on segments which helps to resist slipping and aid in assembly

Membrane analysis performed by Heyman [23] demonstrates that, under a radially symmetric load case, the principal stress directions align with the hoop and strip direction of the conoids. For shells and masonry, it is desirable that the interface between segments be aligned to these directions as that results in minimal shear stress transfer, minimizing the risk of slippage. As such, the segmentation of the conoids of the fan vault can be performed along the hoop and strip directions. This results in a highly repetitive and simple segmentation plan. While it is recognized that this highly idealized radially symmetric loading differs from the one stemming from the actual loads from the spandrel due to radial symmetry being limited to a single conoid in the realised geometry [22]–in fact, even under its own self-weight the spandrel will cause uneven loading on the conoids–any segmentation plan will only be optimal for a certain load case combination and pattern. Considering the advantages that the simpler segmentation plan has for reuse and repeatability, it was selected as the basis for the segmented fan concrete shell flooring system. This difference between the idealised uniform radial loading and the true loading will result in friction and shear forces developing at the interfaces which must be resisted. Such shear forces also occur for other asymmetrical floor loads. This is resolved through the inclusion of shear keys which also has the added benefit of aiding in the assembly process by aligning segments together. The shear keys are formed such that the upper segments have protrusions that rest on the lower segments, shown in Fig. 2.

**Fig. 3:**
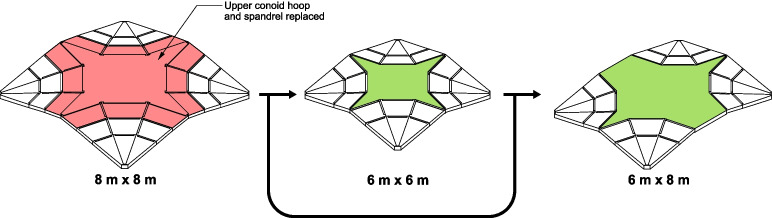
Example reconfiguration of a segmented fan concrete shell by removing components (red) and adding new ones (green)

**Fig. 4:**
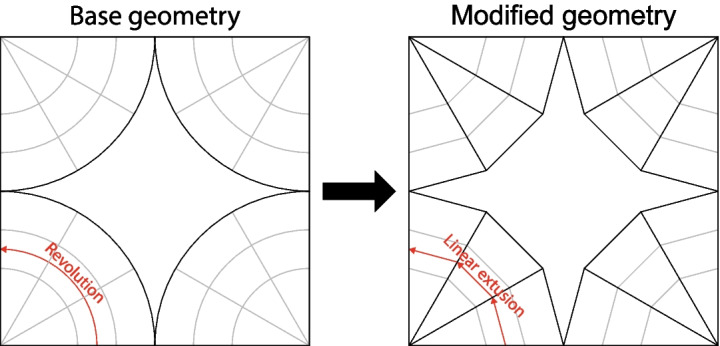
Rationalization of geometry to accommodate fabrication process

#### Reconfiguration

The repeatability and simplicity in the segmentation plan of the fan vault geometry allow for reconfiguration and flexibility in reuse; different distances can be spanned using the same pieces by removing conoid segments and/or adding a new spandrel. Some examples are illustrated in Fig. 3. While structurally a shorter span will be more amenable to reconfiguration, an increase in span distance can potentially be achieved through strengthening and retrofitting of the original segments and interfaces (through the addition of mechanical fasteners to allow for some tensile capacity across interfaces) in order to increase their capacity. Alternatively, the design and form-finding process can be performed for a larger span than what is needed to accommodate future increases in spans. To minimize embodied carbon, maintaining the same span by reusing as many components as possible and reclaiming the spandrel would be ideal. However, by allowing for a degree of flexibility within the system, it incentivizes designers to reuse the building components which increases the chances of enabling circularity for the flooring system. Additionally, as vaulted structures maintain their form and stability through compressive forces at the interfaces between segments, the segmented fan concrete shell can be constructed without any grout or mortar allowing for ease of disassembly for reuse. The simple and repeated pattern of the segmented fan concrete shell also facilitates fabrication due to the lower number of moulds and unique pieces that need to be manufactured and catalogued: fewer casting frames can be made of higher quality, precision, and durability to reuse them multiple times.

#### Fabrication constraints

The fabrication process that will be used for the prototypes introduces constraints that must be considered in the initial design phase. Manufacturing of the prototypes will be carried out using the Automated Robotic Concrete Spraying (ARCS) process [13] which deposits glass fibre reinforced concrete (GFRC) onto a fabric surface bounded by planar wooden formwork. This means that the curvature of the segment boundaries (when viewed from above) must be zero. Modifications to the segmented fan concrete shell were carried out in order to account for this; as opposed to revolving around the column axis, the generating curve profile is instead extruded to fill each strip region (illustrated in Fig. 4). The resulting segment interfaces are therefore contained in planes parallel to the vertical axis, not to the local normal of the shell. This also causes the spandrel to have a polygonal boundary as opposed to an arc boundary. Structurally this causes further deviation from Heyman’s membrane analysis which will result in increased shear forces at the interfaces.

### Finite element analysis methodology

Preliminary analysis of the shell for optimization purposes was performed in Karamba3D [24] – a Grasshopper [25] plugin which can perform linear elastic finite element analysis using shell elements. However, as the interfaces between the segments are to remain ungrouted, this introduces non-linearities and complexities (both due to the concrete material and interlocking compression-only interfaces thanks to the shear keys) which a monolithic finite element analysis using shell elements is not capable of fully capturing [26]. For instance, determining that the structure will fail based on the presence of tensile stresses is too conservative as plastic hinges will form at the interface which will redistribute the loads to other parts of the shell. With respect to buckling, ignoring the effects of the interface results in an overestimate of the load capacity of the structure [27].

In order to address this and find a balance between a detailed non-linear finite element analysis (which would not be compatible with the optimisation process due to its large computational requirements) and a simplistic monolithic and linear-elastic finite element analysis (which does not adequately represent the structure), a novel analysis approach was formulated which combines linear-elastic finite element analysis with a custom joint modelling technique to enable hinging behaviour. The analysis method is detailed in Appendix A.

### Shell form-finding and optimisation

Form-finding was performed using the Wallacei Grasshopper plug-in [28] which implements the NGSA-II multi-objective evolutionary algorithm [29] to converge close to an optimal solution. For the segmented fan concrete shell, two objectives were optimized for: minimised mass and maximised buckling load factor. Minimum mass is desired as this is a good proxy for the embodied carbon of the structure. Because the analysis method (detailed in Section “Finite element analysis methodology” and Appendix A) is expected to produce an overestimate of the buckling load factor, this was also included within the multi-objective optimization in order to provide a range of optimized solutions. This optimized Pareto front can then be used to select the minimum mass solution given a minimum acceptable buckling load factor. The choice of a minimum acceptable buckling load factor depends on the fabrication and assembly tolerances which results in imperfections [30, 31] as well as how much of an overestimate of the buckling load factor the analysis produces. This depends on the amount of segmentation within the shell: smaller amounts of segmentation and interfaces mean that the buckling load factor computed is less overestimated due to being closer to a pure monolithic structure. A more detailed non-linear finite element analysis [32] of select candidates from the final structure can provide an estimate of the amount of overestimation from the optimisation process and can be used to inform the selection of a minimum acceptable buckling load factor.

The parameters of the evolutionary algorithm (i.e., population size, crossover probability, mutation probability, and number of generations) must be appropriately selected. The convergence speed of the optimisation process is affected by the choice of these parameters and, consequently, will affect the results of the algorithm. Converging too rapidly will result in designs which converge too quickly at a local optimum while converging too slowly will require many generations to properly converge. Tuning such parameters is a time-consuming process, with many relying on heuristics [33–35]. For the segmented fan concrete shell, the default settings for the Wallacei plug-in were used for the population size (50), crossover probability (90%), and mutation probability (2%). The number of generations was set to a high number and the algorithm was stopped once convergence was deemed to be obtained (i.e., minimal difference and improvement between generations).

#### Geometry parametrization

The segmented fan concrete shell flooring system was parametrized in order to prepare for form-finding. Firstly, the span of the shell was selected to be 8 m as the maximum design span. The depth is of 800 mm (from the middle of the spandrel to the middle of the base of the conoids), yielding a span-to-depth ratio of 1/10. While this is larger than typical depths of structural floors, the curved profile allows room for services to be integrated within the structural depth (similar to the concrete shell floor detailed in [12]), compensating for the added depth premium. The depth of 800 mm is also comparable to a 300 mm thick flat slab combined with a typical 500 mm height service zone and has been used by other works as a reasonable depth for a curved flooring system [3]. Raised flooring will need to be added to the shell in order to cover the integrated services and also to create a level surface on top of the curved shell. Segmentation was performed every 1 m along the projected length of the conoids in the strip direction and divided into 3 strips per conoid. The exception is the region near the corner which is a single monolithic piece extending 2 m away from the columns, as it is envisioned that the minimum span for a flooring system will not be less than 4 m.

**Fig. 5:**
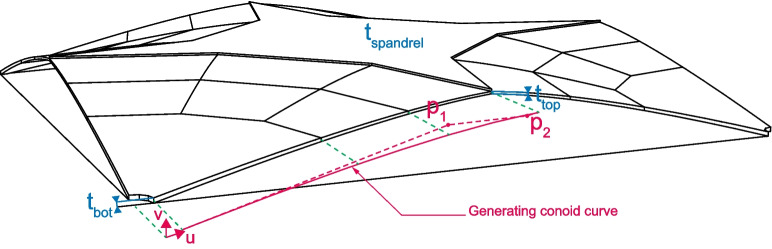
Parametric geometry model of the segmented fan concrete shell flooring system

The thrust surface is set as the medial surface of the vault. The geometry can be defined by setting the midline curve of the conoids and the thicknesses at various locations, totalling 7 parameters. It was determined that a planar Bézier curve with two intermediate points and thicknesses defined at the base of the conoid, the top of the conoid, and the spandrel provides sufficient resolution while not encumbering the form-finding process with excessive parameters. The extremity points are fixed, to link the column and the spandrel. For the thickness in between the top and bottom conoid thicknesses, a linear interpolation was used. In addition, all thickness values are constrained to be within 20 mm to 100 mm. The lower bound of 20 mm is set by fabrication constraints, as it would correspond to a 10 mm thick shear key. The upper bound of 100 mm is set higher than for a common shell thickness-to-span ratio of 1:00, which is 80 mm here, and for a common slab thickness-to-span ratio of 1:30, which is 100 mm here, as the spandrel as a diagonal span of 3.2 m between the conoids. A schematic of the parametrized geometry is shown in Fig. 5, with its 7 parameters (3 thicknesses $t_{bot}$, $t_{top}$, and $t_{spandrel}$, and 4 UV-coordinates $p_{1u}$, $p_{1v}$, $p_{2u}$, and $p_{2v}$). The steel ties are set as a constant M24 steel bar designed to resist the horizontal thrust forces based on preliminary analysis of typical thrust forces occurring in such spans. Including the steel tie area as another parameter would unnecessarily increase the search space for optimization purposes while yielding little benefits as steel bars typically come in discrete sizes.

**Fig. 6:**
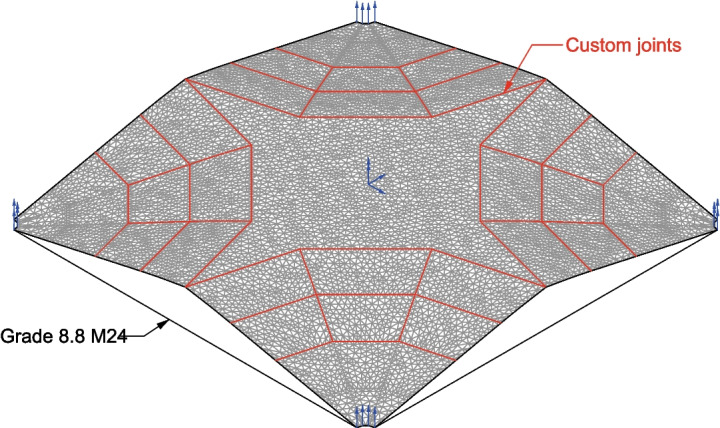
Karamba3D finite element model used for form-finding showing locations of custom joints (red) and restraints (blue)

#### Model statics system

Computation of the buckling load factor objective value is performed by finite element analysis using Karamba3D using the method described in Section “Finite element analysis methodology” and Appendix A using custom joints. A representative model is shown in Fig. 6. Nodes in the corner support were restrained vertically and allowed to translate in the XY plane freely. This is to allow the steel ties which are connected to the corners to be engaged. To prevent the whole structural model from rigid body motions, the node in the middle of the spandrel area is constrained from translating in the XY plane and from rotating about the vertical axis. This does not affect any of the structural results except for the buckling load factor analysis which cannot be properly performed with rigid body degrees-of-freedoms present.

A load combination including the self-weight, an imposed dead load of 1.0 kPa, and a live load of 1.5 kPa (selected as it is more representative of actual loads experienced in office type buildings [36]) was used. The live load was applied over the entire projected area as it was found to likely be the governing load pattern from a preliminary analysis. When other area loadings were applied which can produce a lower buckling load factor depending on the geometry (e.g., a live loading applied only to one half), the difference was minor enough that it did not warrant the inclusion of other load combinations in the optimisation process. Instead, these load combinations should be analysed for the optimised solutions that the algorithm produces. Based on mesh sensitivity analysis, a coarse triangular mesh size of 100 mm was selected, and the loads were applied over 10 load steps. While better convergence was seen with a finer mesh size, the selected mesh density provided a balance of computational speed and accuracy for form-finding which requires the computation of numerous models and geometries. Each model took approximately 2 min to run on the computer setup used.

#### Material properties and limits

Two materials will be used to construct the segmented fan concrete shell: one for the spandrel and one for the conoids. For the unreinforced flat spandrel, a conventional concrete mix with a target characteristic strength of 30 MPa at 28 days was used. For the conoids, the automated robotic concrete spraying (ARCS) process [13] was used to fabricate them as they had curvature and variable thickness. The process involves spraying glass fibre reinforced concrete (GFRC) onto a curved surface bounded by a wooden frame. Typical material properties of components fabricated using ARCS are provided in [13] and were used for the analysis and form-finding. To simplify analysis at the form-finding stage, all segments were modelled using the GFRC material (of which most of the shell except the spandrel consists). Material properties of the GFRC and steel ties used are listed in Table 1.

**Table 1:** Material properties used for form-finding

Properties	GFRC$^{1}$	Steel
Young’s modulus [GPa]	19.9	210
Poisson ratio	0.2	0.3
Weight [kg/m^3^]	2000	8000

$$^{1}$$Obtained from [13]

**Table 2:** ULS requirements considered for form-finding

ULS requirements	Characteristic strength value	Material partial factor
Steel tie util.	830 MPa	1.15
GFRC tensile util.$^{1}$	5.74 MPa	1.5
GFRC compressive util.$^{1}$	31.2 MPa	1.5

$$^{1}$$Obtained from [13]

In addition to obtaining the buckling load factor from the analysis, solutions are constrained and only considered valid if they pass ULS strength requirements. This includes 1) the steel tie utilization, 2) the GFRC tensile utilization, and 3) the GFRC compressive utilization for which the limiting values are listed in Table 2. For the stresses within the shell, the 95th percentile values were used in order to exclude areas of stress concentration in the analysis which is an artefact of the finite element meshing and will redistribute in reality through plastic behaviour, thanks to the ductility provided by the glass fibres.

#### Results

**Fig. 7:**
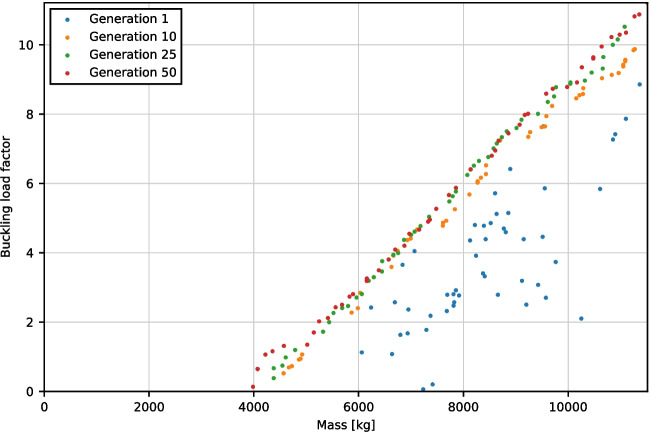
Objective values of the population within select generations from the optimization process

The optimization yields a family of Pareto front solutions which presents the minimum mass geometry given a minimum acceptable buckling load factor under the applied load combination. The performance of select generations is shown in Fig. 7. It can be seen that the population converges onto the approximate optimal Pareto front quite quickly, within 10 generations. This suggests that future optimization and form-finding may be able to be carried out much faster by reducing the overall number of generations. The full optimisation process took approximately 1 week of computation time. Much of this time was spent early in the process whereby inadmissible candidates (i.e., violating ULS requirements) were excluded and not added to the pool of accepted candidates in the generation, thereby requiring additional model generation and computation to reach the pool size of 50.

**Fig. 8:**
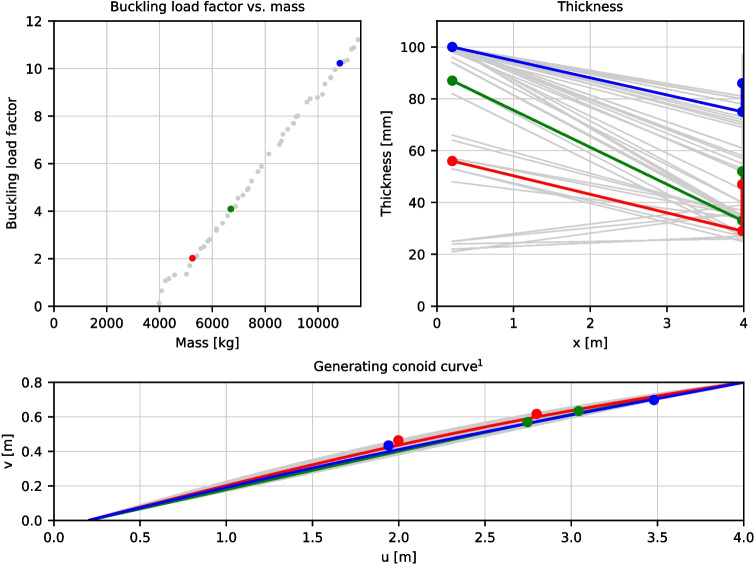
Details regarding the population at the final generation of the optimization algorithm

The results of the last generation are shown in Fig. 8, with candidates with minimum acceptable buckling load factors of 2, 4, and 10 shown in red, green, and blue respectively. Overall, most candidates have similar curve profiles, being a shallow concave curve. This is to be expected given the thinness of the shell and shallowness from the restricted depth: the thrust surface is bounded by the cross-sections at the ends of the conoid and, combined with the small weight of the conoid segments, leads to a low curvature for the thrust surface. The main differences arise from the thickness, where higher buckling load factors can be achieved by increasing the overall mass. In general, optimized solutions have a higher conoid thickness at the base compared to the top combined with an increase in spandrel thickness. This is akin to the effect that a pendant has on traditional gothic fan vaults; by having a large enough spandrel weight, the compressive forces exerted on the conoids are increased and, with sufficiently large values, tensile stresses in the hoop directions are eliminated [23].

A minimum acceptable buckling factor of 10 was chosen to accommodate for the non-linearities unaccounted for by the analysis method as well as any fabrication and assembly imperfections (which can have a significant impact on the performance and capacity of segmented vaulted structures [30–32]). The objective values and the parameters of the selected candidate (along with those with minimum buckling load factors of 2 and 4) are listed in Table 3.

**Table 3:** Properties of the selected full-scale candidate from the optimisation process

	Values		
Minimum buckling load factor	2	4	10
Objective values			
Mass$^{1}$	5.25 t (82.0 kg/m^2^)	6.70 t (105 kg/m^2^)	10.8 t (169 kg/m^2^)
Buckling load factor	2.02	4.09	10.2
Parameters			
Conoid thickness – bottom [$t_{bot}$]	56 mm	87 mm	100 mm
Conoid thickness – top [$t_{top}$]	29 mm	33 mm	75 mm
Spandrel thickness [$t_{spandrel}$]	47 mm	52 mm	86 mm
Bézier point 1$^{2}$ [$p_1$]	(2.00, 0.46) m	(2.75, 0.57) m	(1.94, 0.43) m
Bézier point 2$^{2}$ [$p_2$]	(2.79, 0.62) m	(3.04, 0.64) m	(3.48, 0.70) m
Maximum ULS utilization			
GFRC – compressive	30.2% (6.28 MPa)	28.6% (5.94 MPa)	23.7% (4.93 MPa)
GFRC – tensile	90.1% (3.45 MPa)	95.8% (3.67 MPa)	87.3% (3.34 MPa)
Steel ties	64.3% (464 MPa)	62.3% (449 MPa)	67.7% (489 MPa)

$^1$Calculated using uniform GFRC weight of 2000 kg/m^3^

$^2$U, V coordinate system shown in Fig. 5

As can be seen, the selected design has a thickness that varies from 100 mm to 75 mm within the conoid and has a jump in the spandrel thickness to 86 mm. The Bézier curve profile concaves downwards slightly as expected from the shell. In addition, overall ULS material utilization remains low, with the tensile stresses in the concrete governing. Again, this is similar to masonry structures where the compressive stresses are quite low compared to the capacity of the material itself. For thin shell structures such as these, the utilization becomes slightly higher due to the lower cross-sectional area but remains well within the limits. Under localised point loads, higher tensile stresses arising from localised bending and punching shear will occur. In such cases, membrane action throughout the shell will help to reduce these localised tensile bending stresses. However, further analysis and checks will be required to design against local failure under concentrated loads. For the shell, the magnitude of concentrated loads applicable for typical flooring ($Q_k=2.7~{kN}$ as per BS EN 1991-1-1:2002 [37]) is not expected to govern over the global buckling failure mode induced by area loads.

In the optimisation algorithm, the stress utilisations are treated merely as constraints: candidates that have utilisation greater than 100% are excluded. Including this stress utilisation in the optimisation process would allow for a more optimised design which increases overall material strength utilisation. For example, maximising the mean GFRC material utilisation on top of maximising the buckling load factor and minimising mass would provide insights into how much extra material is ‘wasted’ (with respect to material utilisation). This would enable the design to fully leverage the fabrication process to fabricate a variable-thickness shell that places just enough material where needed. However, this would add another dimension to the selection of an optimal candidate: rather than a simple 2-parameter Pareto front (evident in Fig. 7), a 3-parameter Pareto surface is created. In addition, the extra computational power required to perform this analysis may prove to be unnecessary as such shells are likely to be governed by stability and buckling behaviour as opposed to their material capacity.

## Fabrication and assembly of scale prototypes

Based on the form-found design, quarter-scale prototypes of the segmented fan concrete shell flooring system were fabricated in order to assess their viability and to determine any limitations and shortcomings of the system concerning physical constraints and manufacturing processes. These prototypes measured 2 m by 2 m in span and have depths of 200 mm. All dimensions were scaled to a quarter of the full-scale design except for the thicknesses which were scaled only by half. This choice to only scale the thickness by half is for two reasons. Firstly, the volume and dead weight of the shell decreases at a greater rate compared to the decrease in span length. As such, the thickness must be increased in order to compensate for the scaled-down geometry for stress reasons. While the stability of the shell under its own self-weight is maintained if scaled uniformly, the added self-weight will also aid in counteracting any imbalanced loading that will inevitably be experienced during assembly and instrumentation. Secondly, manufacturing tolerances will have a much bigger effect the thinner the shells are–a minimum scaled thickness of 19 mm would have been too sensitive to the fabrication process’ tolerances, particularly in forming the shear keys that are only half the thickness of the shell and are therefore kept to at least 10 mm as experienced during the development of the fabrication process.

**Fig. 9:**
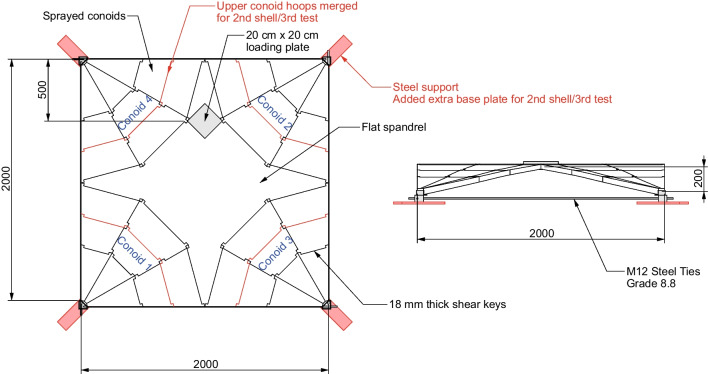
Diagram of quarter scale prototype tests, with variations and modifications between sets highlighted in red with units in millimetres

Two shells were fabricated with differing segmentation plans in order to investigate the effects of the number of segmentations on the overall structural behaviour. The main difference between the two shells is the segmentation of the hoops: in the first shell, the conoids are split into three segments along the strip direction while in the second shell, the upper two conoid hoops are merged. The first shell was tested multiple times as the sprayed conoid segment experienced little damage between tests–only a new spandrel was installed due to cracking and fracture. A schematic of the quarter scale prototype, as well as the locations of the loading plate, are shown in Fig. 9. Variations between the two shells are highlighted in red.

Fabrication of the concrete segments can be divided into two stages: spraying of the conoids using GFRC and casting of the flat spandrel using a conventional concrete mix. Spraying the spandrel was not performed as it was unnecessary due to its flat profile and allows for the use of larger aggregates (thereby reducing the embodied carbon density of the concrete mix per unit volume). Preliminary finite element analysis of the tests demonstrated that an unreinforced spandrel will experience localised cracking due to the concentrated loading, but that this will not greatly affect the overall failure mode of global buckling. In addition to the concrete segments, steel corner supports constructed from angles were made onto which the concrete segments simply rest with no padding or mechanical connection. M12 threaded steel ties were bolted to steel corner supports which the concrete segments rest on in order to resolve the horizontal thrust.

**Fig. 10:**
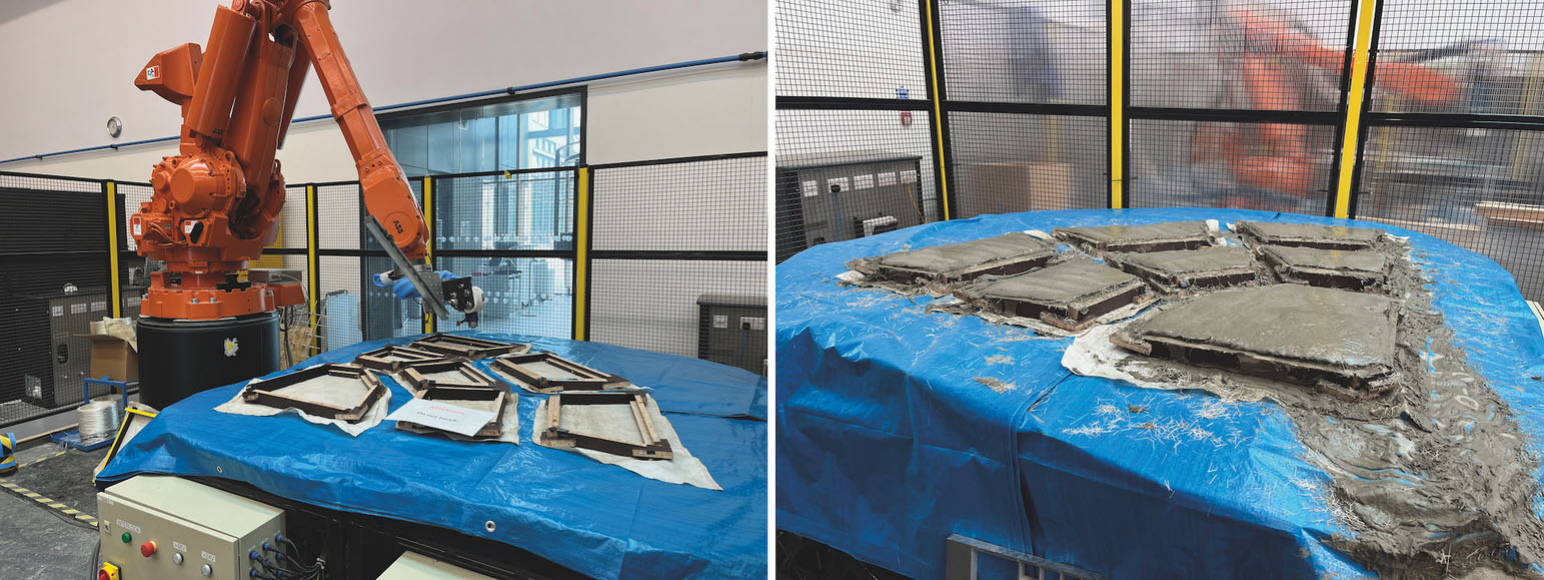
Spraying of a quarter of the conoid segments for the 1st shell prior to (left) and after (right) the GFRC is deposited

### Robotic spraying of conoid segments

The conoid segments were fabricated using the Automated Robotic Concrete Spraying (ARCS) process [13], shown in Fig. 10. This fabrication process consists of two parts: the actuated pin-bed which allows for a desired curved surface to be created and a concrete sprayer which is controlled by a robotic arm that then deposits the GFRC onto the target curved surface. The target spraying area is bounded by a wooden frame constructed out of 18 mm thick phenolic plywood with the shapes cut out using a CAD-guided CNC router. These wooden frames also contain the negative shapes of the shear keys which, when sprayed into, form them. Each conoid quarter was sprayed together during one spray session and demoulded the next day. This limitation on the amount that could be sprayed at once was mainly due to the limited area of the pin-bed mould and the reach of the robotic arm.

The GFRC sprayed onto the wooden frames consists of a cement slurry which is pumped and aerated by the machine, and combined at the end with chopped alkali-resistant glass fibres. The recommended mix from the manufacturer of the concrete sprayer machine (Power-Sprays) was used. This was due to the sensitivity of the sprayer to the cement slurry’s rheological properties and the additives used. Further testing should be performed in order to investigate how to tune the mix to reduce the comparatively high cement content (and embodied carbon density) per unit volume. Quantities of the materials used within the GFRC mix are listed in Table 4. The nominal weight of the mix was found to be 2000 kg/m$^3$.

**Table 4:** Material quantities used for GFRC spraying per meter cubed

Material	Quantity
CEM II cement	795 kg
Kiln-dried sand [max 1 mm agg.]	795 kg
Polycure [curing agent]	80 L
Flowaid [super plasticiser]	4 L
Pumpaid [pumping aid]	4 L
Water$^{1}$	223 L
AR glass fibre$^{2}$	5%

$^{1}$Adjusted to achieve the desired slump of 3 to 4 based on BS EN 1170-1:1998

$^{2}$Fibres chopped to 25 mm at spraying head

**Fig. 11:**
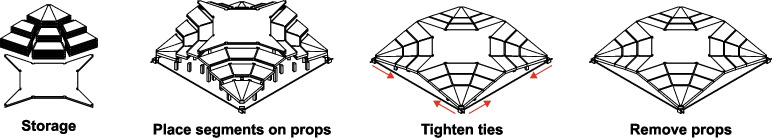
Assembly process of the segmented fan concrete shell, proceeding from left to right

The trajectory planning approach for ARCS involves slicing the shell with curved slices [38]. As spraying was performed in layers, the fibres were deposited and oriented randomly but with the plane of the layers (i.e., generally aligned with the direction of membrane stresses within the shell). From previous investigations [13], it was observed that the achieved thickness of the shell deviates near the boundary regions due to excessive connecting paths and limits in the acceleration of the robotic arm for turning. As such, the thickness of the sprayed material for the smaller upper conoid segments (which have a small ratio of surface area to boundary length) was greater than what was designed. Excess material was removed manually and a thickness correction factor of 75% was applied for later sprayings of the upper hoop segments of the 1st shell. This was found to not be an issue for the 2nd shell due to the larger segments, and the correction factor was removed.

### Fabrication of flat spandrel

The flat spandrel was cast using a conventional concrete mix designed using guidance from [39] to achieve high workability (to ensure a good finish on the shear key interfaces) and a characteristic 28 days strength of 30 MPa. This yields a water-cement ratio of 0.53 and a cement:sand:gravel mass ratio of 1:1.14:3.09. A maximum aggregate size of 10 mm was used for the gravel. Similar to the conoids, the formwork of the spandrel contains negative shapes of the shear keys as formers. No reinforcement was included in the spandrel as it is designed to be held in compression through the arch action of the shell. However, this yields a potentially brittle form for handling, transportation, and storage purposes. As such, the addition of minimum reinforcing bars or other reinforcements (e.g., glass fibres, steel fibres, etc.) may be required for a full-scale structure outside the controlled laboratory environment.

### Assembly

Assembly of the shell proceeds akin to a traditional masonry vault: the segments are placed in their correct locations with the use of falsework which is only decentered once the entire structure is completed. This is necessary as the shell’s stability relies on membrane action and is only truly stable once fully assembled–a condition which is even more important for ungrouted segmented vaulted structures that cannot rely on mortar strength. However, compared to tile vaults, the segments used for the segmented fan concrete shell flooring system are relatively large. As such, there is no need to create an intricate wooden falsework; here, the use of props is sufficient to place the segments at their appropriate height and location. A similar strategy was successfully used previously with another segmented concrete shell flooring system consisting of nine large segments [12].

**Fig. 12:**
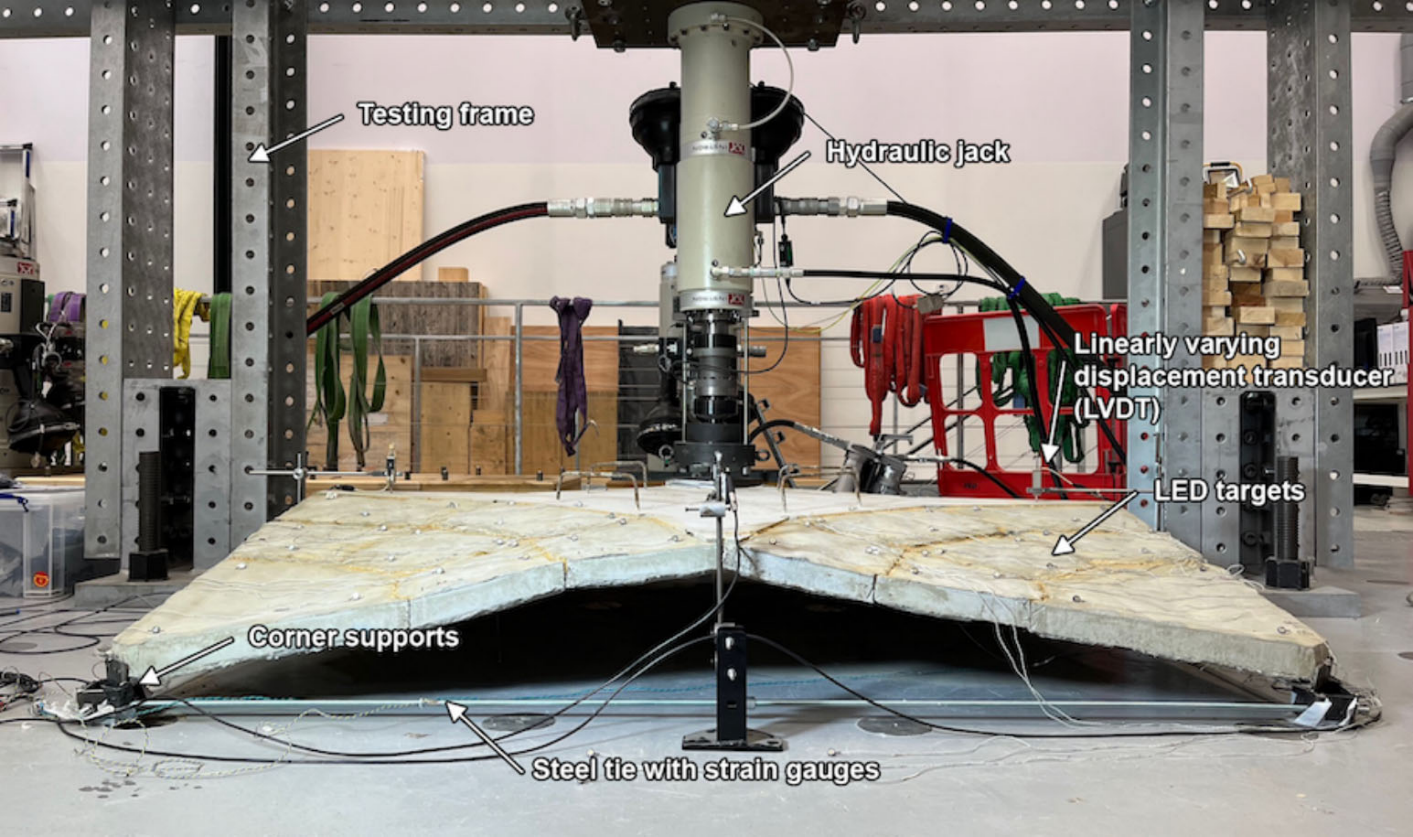
Assembled MN1 shell prior to testing

The assembly process is illustrated in Fig. 11. First, the segments are placed on wooden props and adjusted to their correct height. The steel ties are then tightened in order to engage arch action. Lastly, the props can be removed one at a time. Disassembly of the structure proceeds in the reverse order of the assembly process. This was successfully performed for Shell 1 which was disassembled and reassembled prior to the first test. The disassembly process was found to be much faster compared to the assembly process as segments need not be aligned and placed in their correct position and heights. In addition to this, Shell 1 was reassembled a second time with a new spandrel for further load testing after the initial test, demonstrating that reuse is possible by combining old and new components with no extra complexity. While not as direct of an assembly process compared to conventional precast slabs, the use of discrete props presents an improvement to traditional funicular and masonry construction which typically involves the construction of a geometrically complex secondary support structure.

Due to tolerance issues, the fabricated segments did not fit perfectly together. This was caused by small deviations in the fabrication of the wooden frame and shearing deformations from the relatively soft corner of the frame, amounting to a maximum of 10 mm. Using a more precise and stiffer steel formwork would enable higher accuracy while also enabling reuse, as the wooden formwork warped and degraded after several casts. In order to allow the shell to stand, a fill material was added in between the segments once they were placed on the props which helped to close the gaps and allow contact to form while still allowing for disassembly. For the first shell, fine sand was used to fill the gaps for the first test (MN1) and mortar was used for the second test (MN1M). As the strength of the mortar was not of great concern (rather its role as a filler material was its main purpose), MN1M was decentered merely a day after the mortar was applied and then tested within a week resulting in an overall weaker mortar which has not developed all of its strength. For the second shell, the deviations caused by tolerance issues were too great and fine sand could not adequately be used. As such, only mortar was used to fill the gaps for the last shell and test (MN2M). This use of mortar does limit the disassembly potential of the prototype shells. However, it was performed in order to proceed with the assembly and further testing of the fabricated pieces. When scaled up in an off-site prefabrication plant process, these deviations and tolerance issues are not expected to have as large of an impact compared to what they have on these quarter-scale prototypes. For example, a typical formwork dimension deviation for precast concrete of 2.5 mm [40] is comparatively smaller for the full-scale design of the segmented fan concrete shell compared to the scale prototypes where the smallest segments are less than 500 mm in side lengths.

Once assembled, 3D scans were taken of the shells which were used to measure their thicknesses and for analysis purposes. The results of this are provided in Appendix B.

## Structural load testing

### Methodology

Testing of the shell was performed at the National Research Facility for Infrastructure Sensing at the University of Cambridge. A total of three tests were carried out, two using the first set of sprayed conoids (MN1 and MN1M) and one using the second set of sprayed conoids (MN2M). In addition to differences in the segmentation of the conoids, the tests varied with the material used to fill the gaps: either sand or mortar (the latter is denoted by the addition of -M at the end of the label). An asymmetric point loading was applied on the shell (offset 500 mm towards an edge), whose purpose is to induce an instability failure rather than a punching failure which would have been induced by a central point load. During the test, displacements of the jack and the segments were monitored using an LED tracking system. A displacement-controlled loading was applied at a slow rate of 1 mm/min. In addition, the strains of the steel ties were recorded using strain gauges and displacements at the middle edges of the shells were measured using linearly varying displacement transducers (LVDTs) (Fig. 12).

**Table 5:** Mean material properties from tests

	Seg.	# Tests	Compression cube		Bending prism		
			$E_{c,comp}$	$f'_c$	$E_{c,bend}$	$f_{t, lop}$	$f_{t, ult}$
			[GPa]	[MPa]	[GPa]	[MPa]	[MPa]
Shell 1							
28 days	1	4/2	34.9	36.4	10.1	9.12	11.1
	2	4/2	31.4	38.9	13.6	8.88	11.4
	3	4/2	20.6	30.1	8.54	9.07	11.1
	4	4/2	11.6	26.6	6.61	7.08	8.48
MN1	1	4/2	42.4	44.0	11.2	8.23	9.99
(test day)	2	4/2	30.5	43.2	10.3	8.66	10.3
	3	4/2	15.1	26.7	11.8	9.79	12.3
	4	4/2	27.5	20.4	8.20	7.47	8.12
	S	20	33$^{2}$	41.4	–	–	–
MN1M$^{1}$	S	10	33$^{2}$	45.4	–	–	–
Shell 2$^{3}$							
28 days	1	2/1	14.4	41.0	8.81	9.53	13.5
	2	2/1	12.7	30.1	4.74	8.68	10.4
	3	2/1	0.53	28.6	7.06	7.71	10.1
*26 days*	4	2/1	25.0	42.2	11.9	9.79	9.86
MN2M	1	6/3	6.89	34.4	11.4	10.5	13.1
*(test day)*	2	6/3	6.28	27.3	8.24	7.63	9.43
	3	6/3	9.84	34.3	12.0	8.75	11.9
	4	6/3	14.1	43.4	12.3	9.02	12.1
	S	10	32$^{2}$	33.4	–	–	–

Conoid numbering matches Fig. 9

$^{1}$MN1M did not have sprayed material tests

$^{2}$Spandrel stiffnesses interpolated based on BS:EN1992-1-1

$^{3}$Premature spalling and localised crushing of cube specimens observed

### Material tests

For each set of sprayed segments, a set of material testing samples was fabricated by spraying a 40 mm thick flat panel and cutting out prisms and cubes [13]. Bending tests were performed on four prisms measuring 40 mm thick and 50 mm wide tested in four-point bending over a 300 mm span as per BS EN 1170-5:1998 [41]. Two values were extracted from the tests: the cracking stress ($f_{t,lop}$) which is the stress at the extreme tensile fibre at which non-linearities due to cracking first appear and the ultimate tensile strength ($f_{t,ult}$) which is the maximum tensile stress at the extreme tensile fibre. In addition, eight 40 mm cubes were tested in pure compression (adapted from compression tests of mortar prisms tested under 40 mm by 40 mm square loading plates as per BS EN 1015-11:2019 [42]), ensuring that the loading is parallel to the plane of the sprayed fibre (i.e., same loading direction as would be experienced by the sprayed conoid segments). From this test, the stiffness of the material ($E_c$) and the ultimate compressive strength ($f'_c$) were obtained by taking the tangent stiffness and the maximum stress of the loading curves. In addition to the GFRC tests, standard 100 mm concrete cubes were cast and tested in compression for each flat spandrel cast. The ultimate compressive stress ($f'c$) was obtained from the tests while the stiffness of the spandrel was interpolated based on BS EN 1992-1-1 [43]. The tests were spread out such that values were obtained for 28 days after the cast and on the same days as the tests, except for shell MN1M which did not have any separate material tests performed for the conoids.

**Fig. 13:**
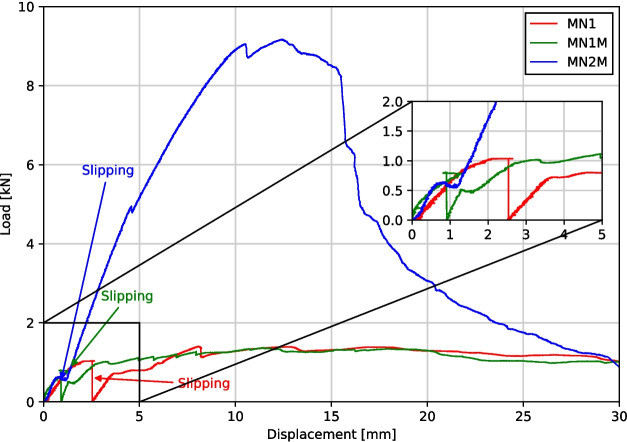
Load against displacement of jack for the three shell tests

**Fig. 14:**
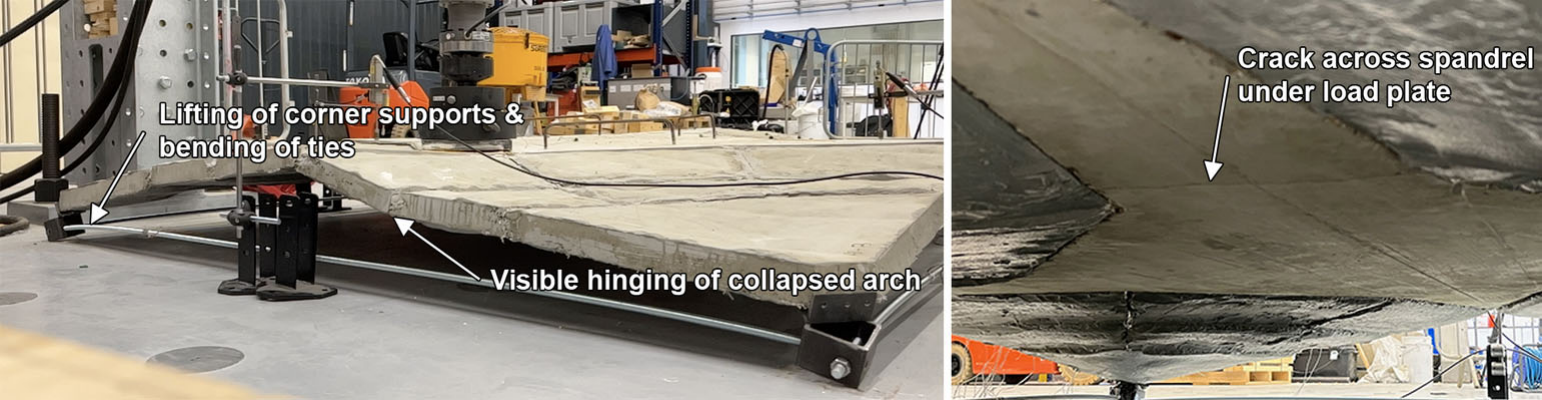
Visible support rotation and tie bending accompanied by a visible collapse of the arch (left) and cracking of the spandrel underneath the load plate (right) observed during the MN1M test

The mean values of the properties are listed in Table 5. Note from the values listed, the variability of the material properties, especially the conoid material’s stiffnesses, is quite large, despite the same GFRC mix being sprayed. Such variability is likely from the manual mixing and machine calibration process involved: for each spraying session, the machine is recalibrated manually to obtain a target glass fibre content and the cement slurry’s water content is adjusted to achieve a desirable slump. In addition, premature spalling and localised crushing were prevalent within the cube samples for Shell 2, leading to unrealistically low compression stiffness values, further exacerbated by the displacement tracker targets spalling off the cover (for example, the 0.53 GPa stiffness value for segment 3’s material samples were a result of this latter issue). This was likely due to fabrication issues of the test panels which occurred with Shell 2; it was observed that the cement slurry had started to set as a longer time was taken between spraying the conoid segments and the test panel compared to with Shell 1. This resulted in a mean difference of 49.2% between the 28-day compressive stiffnesses of the two shells, despite identical mix designs being used. Based on this, the results of the material tests on sprayed samples of Shell 2 are not expected to be representative of the actual sprayed conoids.

### Results

MN1 and MN1M were tested 28 days after the spandrel was cast while MN2M was tested 27 days after. The displacement of the jack is plotted against the load applied for all three tests in Fig. 13. The displacement is set at zero for when the load starts to be applied (i.e., when the jack starts to touch the loading plate on the shells).

#### MN1 and MN1M

The MN1 and MN1M tests both exhibit very similar responses; an initial linear response is observed with a softening and further ductile behaviour. This was accompanied by cracking near the load plate at the spandrel early in the loading, which demonstrates hinge formation. A maximum load of 1.40 kN and 1.37 kN was measured for MN1 and MN1M respectively. This is equivalent to merely 35% of the self-weight of the shells. No visible signs of damage and cracks were observed in the conoid segments throughout the test.

**Fig. 15:**
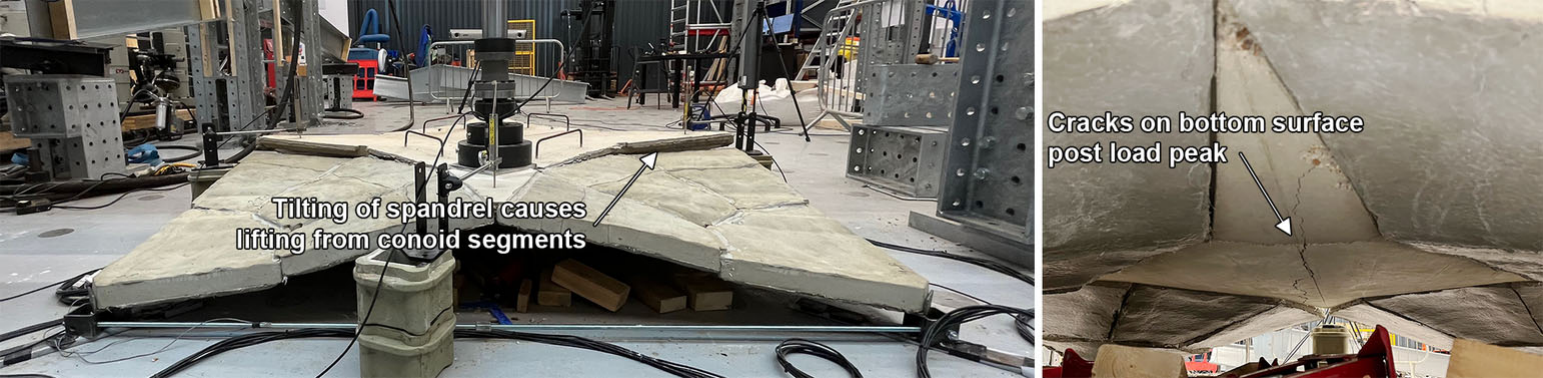
Lifting of spandrel from conoid segments due to tilting (left) and cracking of the spandrel underneath (right) observed during the MN2M test

**Fig. 16:**
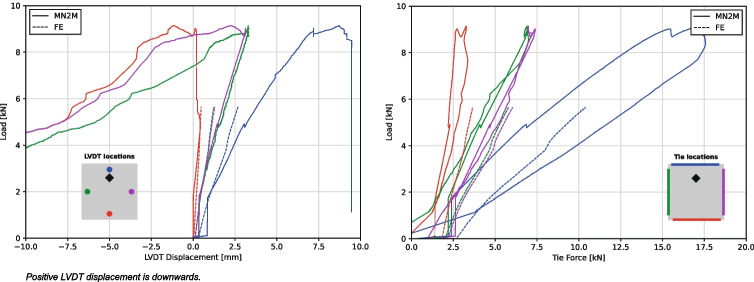
LVDT displacements (left) and tie forces (right) for MN2M from test and FE analysis

For both MN1 and MN1M, it was observed that a large slip between segments caused a drop in the applied load of the jack. This is to be expected for MN1 as the fine sand used as fill had minimal shear strength; as the load increases and the stresses transferred across the interface increase, the interfaces will slip. However, this does not cause collapse as the shear keys prevent further slips from occurring. For MN1M, the low strength of the mortar will act similarly to the fine sand used which also has minimal shear strength. Both tests exhibit similar stiffnesses and final collapse loads. This suggests that the fill material (sand or mortar) does not have a significant impact on the structural behaviour of the shell.

During the assembly stage and testing of both MN1 and MN1M, the ties experienced significant bending and the corner supports exhibited large rotations, shown for MN1M in Fig. 14. This is a direct consequence of the corner supports being too light as well as the fact that the thrust of the shell is not perfectly aligned with the resultant forces of the ties. This affected the behaviour of the shells during the test in two ways: 1) the corner supports allow for greater displacements of the corner segments due to the additional rotation, resulting in a less stiff response compared to a structure with non-rotating supports and 2) the failure of the shell is primarily caused by the corner elements slowly slipping on the rotated corner supports, spreading the shell and inhibiting arch action. In addition to this, the tie strains measured from strain gauges applied on one side of the ties included bending and did not properly reflect the average axial strain of the ties. For the next test (MN2M), larger corner supports were fabricated to minimise the rotation and lifting of the corner supports. Moreover, an additional strain gauge was added to each tie opposite the original strain gauge in order to allow the calculation of the average axial strain in the tie.

After the peak of the tests has been reached, the shell exhibits ductile behaviour. Visually, it can be seen that most of the arches within the shell have collapsed (i.e., significant deformations are present and interfaces between segments showed visible hinging rotations). However, the shell is able to resist further jack displacement as each of the segments is still able to rest on each other, allowing the collapsed individual arches to remain intact as a full shell.

#### MN2M

For MN2M, a significantly higher collapse load of 9.17 kN was measured, equivalent to 231% of the self-weight of the shell. From Fig. 13, the shell can be seen to exhibit an almost linear response past the initial slipping phase, with a softening response caused by cracking of the spandrel at 4.5 kN. The shell then exhibits some non-linear response in the form of softening and a decrease in load. No visible cracks or damage were observed up until 4.5 kN, where a small crack running across the spandrel was observed (shown in Fig. 15). Notably, the crack that was found directly under the load plate in MN1 and MN1M at low loads was not observed in MN2M. Similar to MN1 and MN1M, ductile behaviour can be observed past the peak load. It is at this ductile phase that large cracks start to appear in the bottom surface of the spandrel: a new one under the loading plate and an enlarging of the crack running from the spandrel corner with the load plate to the opposite corner, shown in Fig. 15.

**Fig. 17:**
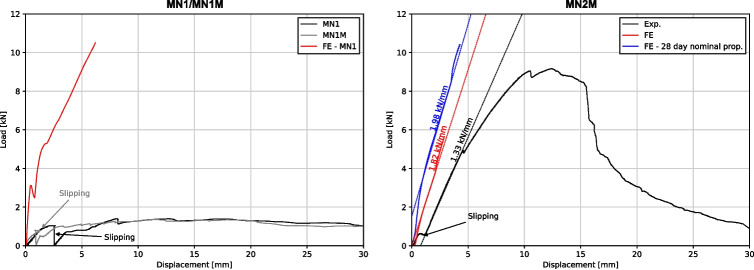
Load displacement curves of load jack from FE analysis plotted alongside experimental results for MN1 and MN1M (left) and MN2M (right)

The tie forces and the displacements of the LVDTs placed at the corners of the spandrels 200 mm away from the edges are shown in Fig. 16 alongside results from FE analysis (which is discussed in Section “Structural numerical analysis”). Similar to the load-displacement behaviour of the jack, the tie forces and LVDT displacements increase almost linearly with the load. Once peaked, the tie forces for all the ties start to decrease until they fall to zero, which correlates with the overall decrease in load applied by the jack. The LVDT displacements display the tilting behaviour of the spandrel; while displacement tracked near the edge closest to the loading plate continues to increase slowly, the other corners of the spandrel start to lift upwards. This suggests that the spandrel is not being supported by all segments any longer, but rather that it is being supported at set points and pivoting around them.

## Structural numerical analysis

Nonlinear finite element analysis (NLFEA) of the scale prototypes was carried out using LS-DYNA. The modelling approach is detailed in [32] and utilizes contact surfaces to allow stresses to transfer between discrete segment parts. Solid tetrahedral elements were used which were meshed from the 3D scanned geometry obtained after assembly (B). A mesh sensitivity analysis demonstrated that an equivalent element size of 15 mm was adequate for convergence. The Continuous Surface Cap Model (CSCM) was used to model the concrete material, with the stiffness and compressive concrete strengths adjusted to match those obtained from material tests. The tensile strength of the CSCM material is lower compared to that observed in the sprayed GFRC due to the lack of fibre reinforcement (approximately 4 times lower compared to test values). However, this was left as is to avoid further parameter tuning of the complex material model and since minimal tensile stresses are observed in the FE analysis within the conoid segments, suggesting that additional tensile capacity will not affect the behaviour of the shell under this highly asymmetric loading meant to induce instability. The cracks that were observed were limited to the spandrel which is a typical unreinforced concrete mixture with low tensile strength, matching the CSCM model well.

Loading was applied in two stages. First, a preloading phase was performed which applies the self-weight of the shell. This allows the contacts to appropriately engage and also models the assembly stage of the shell: the structure supports its self-weight prior to the application of the localised point load. After this preloading phase, a displacement-controlled loading was applied at a variable maximum rate starting at a low 0.01 mm per load step (as contact between the loading plate and the shell is formed) and increasing to 1.00 mm per load step, with LS-DYNA allowed to adjust this based on the rate of convergence of the previous load step.

The corner supports of the shell were treated as rigid bodies due to their much higher stiffness compared to the concrete shell. These corner supports were prevented from rotating and moving vertically, only being allowed to move along the plane of the floor. Tie elements were used to represent the M12 ties in the shell.

### Results

#### MN1 and MN1M

The results of the FE analysis of MN1 and MN1M were almost identical. This is as expected as the geometry and thickness distributions of both shells are very similar, as shown in Fig. 22. As such, only the FE analysis results of MN1 are included here for brevity. Comparison of the tie strains was also excluded as the measured tie strains are affected by the bending strains observed in the ties while the FE model employed tie elements which only resist axial forces.

The load applied by the jack was plotted against the jack displacement from the experimental results alongside results from both MN1 and MN1M in Fig. 17. As can be seen, the two tests exhibit a much less stiff response and also a much lower maximum load capacity compared to the 10.5 kN obtained from the FE analysis. This is to be expected due to the rotation of the corner supports resulting in a lower stiffness as the shell is allowed to spread without fully engaging the axial stiffness of the ties.

To investigate this further, the model was modified to allow rotation in the corner supports by capturing the contact between them and the flat ground. The ties were also changed from tie elements to beam elements, with one end fixed to the support to allow moment transfer while the other side was left pinned. These end conditions mimic the end conditions of the ties in the experiment as one side was threaded through the corner supports while the other was attached by a single bolt. The FE analysis of MN1 performed with support rotations included predicts failure at the self-weight preloading phase, with difficulties in converging when only 57.4% of the self-weight has been applied due to the precarious contact between the corner supports and the corner conoid segments. This demonstrates reliance of the structural capacity and stability on the support conditions; with MN1 and MN1M, the rotating corner supports both reduce the stiffness and maximum load capacity of the shell. The rotation also creates instabilities in the contact surfaces as the corner elements now do not rest well on the supports, creating difficulties for the FE analysis to converge.

#### MN2M

As previously stated, the material tests performed on the test date of shell MN2M exhibited premature failure in the compression tests due to localised spalling and delamination which are specific only to the test panel. As such, the material properties will not be representative of the actual stiffnesses of the conoid segments in the shell. In order to accommodate for this, two different FE models were created based on 1) the material properties taken from the material tests and 2) nominal 28-day material properties of the sprayed GFRC [13].

The load applied by the jack was plotted against the jack displacement from the experimental results alongside the two FE models in Fig. 17. As can be seen, the stiffness observed in the linear portion of the experimental test is quite closely matched by the stiffness of both FE analyses, with a percentage difference of 31.1% and 39.2% for the models with material tests properties and the nominal 28-day material properties respectively. The increased conoid segment stiffness used in the 28-day FE model shows a significant increase in the collapse load of 10.4 kN compared to 5.65 kN, which translates to a percentage difference of 12.6% and 47.5%. As the failure mechanism in the FE analysis is through an instability similar to buckling, it was expected that a higher material stiffness would result in a higher collapse load. Past the load peak, the implicit analysis scheme used has difficulties converging and finding a suitable equilibrium solution. In the actual physical test, this instability will result in a readjustment of the load paths and a slow ductile failure (an effect of the displacement-controlled loading used).

The tie forces and the vertical displacements of the nodes in the first FE model are plotted alongside experimental measurements in Fig. 16. The increases are mostly linear, and the gradients agree well with those measured. For the tie forces, the starting forces applied by the ties in the experiment do not match the forces in the FE model which resists the thrust from gravitational loads. This is due to the manner in which the ties were tightened in the structure; the ties are slowly increased until the shell lifts slightly off of the supports. As the shell starts to settle initially, the initial tie forces will vary and may not exactly match those observed from the FE model.

### Discussion

Due to the aforementioned difficulties with manufacturing tolerances at the interfaces and the rotating support conditions of one of the shells, it is difficult to assess whether the FE analysis methods described in [32] can be used to properly capture the behaviour of the segmented fan concrete shell prototypes. However, these uncertainties present barriers and difficulties for any analysis methods, and as such are challenging to assess regardless of the methods used. Further testing of prototypes will be required in order to improve the test data and limit the number of variables that affect the structural performance of the shell.

Regardless, some trends can be extrapolated from the FE analysis, especially for MN2M where the issue of rotating supports is not present. The collapse load is shown to be sensitive to the stiffness of the conoids themselves, with the model using the more realistic 28-day properties providing a closer match compared to the model using material properties obtained from material tests which failed prematurely. The ductile post-peak behaviour of the shell is difficult to capture using the implicit analysis scheme utilised for the FE analysis. If it is desired to model this, an explicit analysis scheme may be utilised, although this requires further investigation and validation.

The FE analysis of the MN1 and MN1M demonstrated the potential capacity of these shells when no support rotations are allowed. The rotating supports in the experiment significantly reduce the structural capacity of the structure due to increased spreading. Attempts to model this behaviour in the FE analysis resulted in models that were not stable enough to support their own self-weight. In real life, this instability can result in a slip which results in the structure resettling into a stable configuration and being able to resist more load. However, this is difficult to capture numerically in an FE analysis. Regardless, the weak structural stability of MN1 and MN1M is well demonstrated by the low maximum loads of the shells in the experiments compared to both MN2M and the predicted FE analysis (under non-rotating supports).

Due to the rotating supports, not much can be concluded regarding the effects of the segmentation on the structural behaviour of the shells based on the tests. However, some conclusions can be extrapolated from the nonlinear numerical analysis. The load capacity between the models shows similar collapse loads, although Shell 1 exhibits a less stiff response. This is because the collapse mechanism under this asymmetric loading does not rely on the formation of the hinges at the additional interfaces present in Shell 1. Under a different load case–the worst case being a direct point load at the location of the interface–it is expected that the additional interface will result in a drastic drop in load capacity. In addition, a higher number of interfaces will exacerbate any tolerance and fabrication issues which is present–a factor which is difficult to account for numerically in the implicit analysis scheme used [32]. As such, it can be concluded that increased segmentation will affect the structural performance negatively, although further investigation is required to quantify the extent under various load cases.

## Embodied carbon comparison

An embodied carbon comparison between the segmented fan concrete shell flooring system compared to conventional flooring systems is detailed herein. Firstly, it must be stated that the embodied carbon of the shell drops drastically if the segments are reused. With the lack of steel reinforcements inside the segmented fan concrete shell minimising durability concerns, the components can potentially be continuously and indefinitely disassembled and reused. However, such promises of potential reuse in the future are not assured (as demonstrated from previous case studies [44]) and should not be used as an argument to justify a significant embodied carbon premium. Furthermore, it is acknowledged that the following presents merely one facet of sustainability for comparing various structural options. Assembly considerations and labour effort, which adds to the overall energy, cost, and embodied carbon, are outside of the scope of this comparison. Compared to conventional precast slab construction, the segmented fan concrete shell is likely to perform worse in this aspect. However, further work involving large-scale assembly, prototyping, and refinement of the assembly process is required to properly quantify the effects of this. As such, this simplified analysis can be viewed as a means of assessing the sustainability potential of a novel structural system at the early stage of development and to evaluate whether there is potential for further development and work.

The analysis was performed using a *cradle-to-gate* boundary (equivalent to Modules A1 to A3 of the BS EN 15978 [45] life cycle stages). Four flooring systems were selected to be compared: 1) a flat reinforced concrete slab, 2) a voided hollow deck concrete system, 3) a thin-shell concrete flooring system also fabricated using GFRC (the ACORN shell [12]), and 4) the segmented fan concrete shell. Calculations were performed based on a span of 8 m by 8 m using the same ULS load combination used for form-finding purposes. It should be noted that an 8 m by 8 m is not optimal for flat and voided slabs to reduce their embodied carbon effects [46]. However, this span was used for comparison purposes with the segmented fan concrete shell which has been designed for this particular span. Conversely, the ACORN shell was form-found against a 4.5 m by 4.5 m span distance. However, this was not modified as it would require redoing the original form-finding process of the ACORN shell [12]. Furthermore, the false flooring has not been included in the embodied carbon comparison. This is a requirement to create a flat surface for the shells, but is only optionally employed in conventional slab construction depending on building usage and other requirements. For this simplified analysis, it is assumed that all options will utilise false flooring as it is a relatively common system employed in office-type spaces to contain services.

**Fig. 18:**
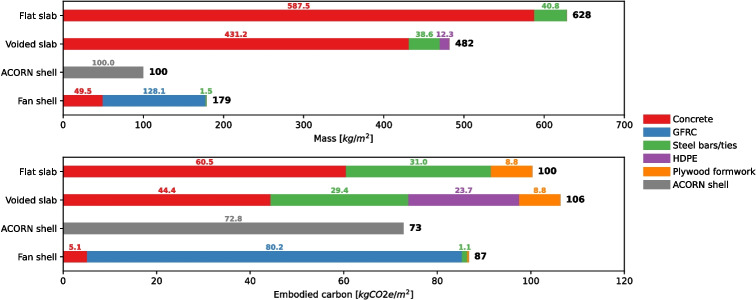
Comparison of mass and embodied carbon of various flooring systems with ACORN data taken from [12]

**Fig. 22:**
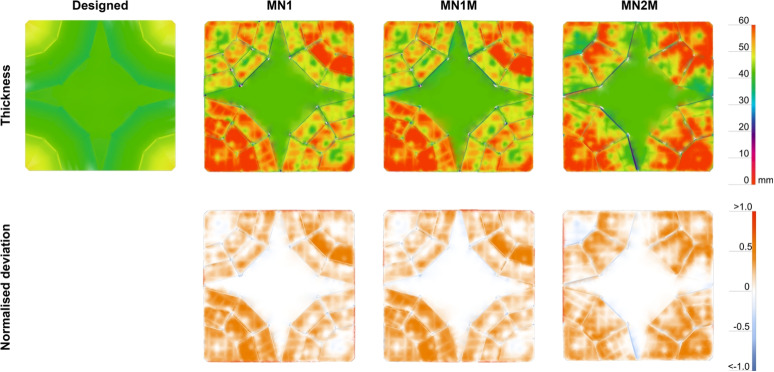
Thickness of designed shells compared to 3D scan data and the deviations from the designed thickness, normalised by the spandrel thickness of 50 mm

The material of the concrete (for the flat slab, voided slab, as well as the flat spandrel) is taken to be C30/37 class concrete with 35% cement replacement. Conventional steel reinforcement is taken to have a yield stress of 500 MPa. Design of the flat slab was carried out as per Eurocode 2 and the voided hollow deck system was designed as per a manufacturer’s recommendations [47]. The high-density polyethylene (HDPE) bubbles used to form the voids were assumed to have a uniform thickness of 5 mm. Raised flooring is excluded from the calculations as it is assumed that all the flooring systems considered will employ it in order to accommodate services. Embodied carbon impacts of the various materials are listed in Table 6 and the total mass and embodied carbon of each flooring system is shown in Fig. 18. The mass and embodied carbon impacts of the ACORN shell were taken directly from [12].

**Table 6:** Embodied carbon factors of materials

Material$^{1}$	Embodied carbon factor	Source
	[kgCO2e/kg]	
***Standard***		
	0.103	[48]
	0.76	[49]
	0.681	[50]
***Voided slab***		
	1.93	[51]
***Segmented fan concrete shell***		
*Individual GFRC components*		
Cement	0.832	[50]
Sand	0.00747	[50]
Polycure FT (curing agent)$^{2}$	1.67	[50]
Flowaid FT (super plasticiser)	1.88	[50]
Pumpaid FT (thixotropic pumping aid)$^{2}$	1.67	[50]
Alkali resistant glass fibre	3.00	[52]
	0.626	

$^{1}$Materials coloured to match those used in Fig. 18

$^{2}$Taken from the average factor of concrete admixtures

From the analysis of embodied carbon of the four flooring systems, it can be seen that the two shells have the least embodied carbon per floor area, with the ACORN shell and the segmented fan concrete shell having a reduction of 27% and 13% compared to the flat slab alternative respectively. These savings are quite modest compared to the mass reduction, having only 15.9% and 28.5% of the mass of the reference flat slab design. The cause of this difference is rooted in the high embodied carbon of the sprayed GFRC mix, mainly due to its high cement content and lack of larger aggregates. Further work to tune the mix to improve its sustainability (e.g., using cement alternatives or reducing the cement content) will allow for a significant reduction in the embodied carbon of the shell. Despite this, the analysis demonstrates that funicular flooring systems are a lightweight and sustainable alternative to conventional flooring systems.

Comparing the ACORN shell and the segmented fan concrete shell, the ACORN shell is shown to be both lighter and to have less embodied carbon. This is to be expected as the ACORN shell is not designed for reconfigurability (although it is compatible with disassembly and reuse [12]). The constrained form-finding and design approach of the segmented fan concrete shell produces a mass premium of 79% and an increase in embodied carbon of 19%. The smaller difference in the embodied carbon density is due to the use of the lower embodied carbon density of the concrete mix of the flat spandrel compared to the ACORN shell which is made entirely from sprayed GFRC.

Regardless, the disassembly, reuse, and reconfiguration potential of the segmented fan concrete shell (demonstrated through the assembly and disassembly of Shell 1) combined with its savings in embodied carbon presents an improvement over the conventional flooring systems analysed. Beyond the flooring system itself, the low mass results in far lower loads on the columns and footing, allowing material and embodied carbon savings elsewhere within the superstructure and substructure of the building. This highlights another advantage of a lighter funicular flooring system outside its disassembly and reuse potential.

When considering reuse and circularity (i.e., Module D of the BS EN 15978 [45] life cycle stages), the potential for further reduction drastically increases, potentially offsetting the majority of the Module A embodied carbon depending on the amount of segments reused, with 72% of it initially coming from reclaimed concrete floor modules. However, promises of future reuse should not be relied upon to justify a higher embodied carbon cost as they may not be realised [44] and are dependent on factors outside of the designer’s control at the end of the building’s lifespan (e.g., social acceptance, state of economics, political climate, etc.). The segmented fan concrete shell however facilitates this reuse option without requiring it to make it a sustainable option, making it a flexible and sustainable flooring system, with potential to reuse all or part of the structural components if designed durably.

If a more sustainable mix is utilised to fabricate the conoid segments (take the C30/37 concrete as a reference), the embodied carbon density of the segmented fan concrete shell drops drastically from 86.4 kgCO2e/m^2^ to 19.4 kgCO2e/m^2^, a 78% reduction. However, the GFRC material presents advantages for fabrication purposes (as it is a necessary component of the robotically sprayed fabrication process to produce variable thickness thin-shell concrete [13, 38]) and the inclusion of glass fibres helps to increase the durability of the components, especially for transportation, storage, and assembly purposes. Regardless, this demonstrates the benefits of the form independent of the material and shows that there is space for further sustainability improvements in the design.

## Conclusions and future work

The proposed segmented fan concrete shell contributes to the search for a materially and carbon-efficient flooring system that is compatible with disassembly, reuse, and reconfiguration. Scale prototypes of the form-found geometry were fabricated and tested in order to evaluate the structural performance of the system and identify any limitations and deficiencies of the proposed system. The work herein adds to the growing literature of thin shell flooring systems as a sustainable alternative and contributes experimental test data that can be built upon.

Compared to the ACORN shell (another form-found thin-shell concrete flooring system), the added constraint of designing for reconfiguration adds a mass and embodied carbon premium to the segmented fan concrete shell flooring system. However, the system remains a more carbon and material-efficient alternative to flooring systems where bending dominates, and the choice to design for reconfiguration merely adds a 19% embodied carbon premium over a comparable segmented shell flooring system. Whether the ability to easily reconfigure the flooring system for future reuse cases is worth this premium should be decided upon by the client, designer, and/or construction parties weighing in other factors of the project.

The load tests highlight the sensitivity of the segmented fan concrete shell system to support conditions as well as fabrication tolerances. In the first set of shell tests, the supports experienced significant rotations which resulted in increased spreading and a drop in the stiffness of the structure. In comparison, the larger corner supports of the second shell test contributed to the drastically higher load capacity and stiffness observed; factors which could not merely be attributed to the differences in segmentation layout. Further testing and prototyping are required to provide further validation of the finite element analysis methodology utilised as well as to isolate fabrication and testing factors that affect the structural performance of the shells.

Further work is required to investigate whether the current dry joint interface with shear keys is the appropriate interface type for shells. Mechanical fasteners may allow for more reliable and predictable force transfer while also being able to be disassembled. Unbonded post-tensioning could also present another viable means of compressing the segments together while being reversible. In addition, further work is required to tune the embodied carbon of the GFRC mix to better translate the material and mass savings into embodied carbon savings. Outside the structural behaviour and sustainability factors, investigations into the fire safety performance of the shell (because of fire-resistance of glass fibres, and by protecting the steel ties), integration of mechanical services (through standard raised floors), as well as acoustics (by breaking the smooth geometry with structural ribs or non-structural acoustic panels) are needed in order to bring the system closer to implementation in practice. The existence of historical precedence of shells as flooring systems in buildings (e.g., Guastavino’s tile vaults, etc.) suggests that these are more than likely solvable design problems rather than barriers that will impede the proposed system from practical viability. Lastly, constructability and assembly considerations must be addressed. Compared to conventional precast slab construction, the assembly process of the segmented fan concrete shell is more involved, leading to higher labour costs and energy usage. The use of discrete props as opposed to a complex support structure already presents an improvement over conventional masonry construction, but falls short of the rapid erection rate of modern precast construction. This may potentially be solved by manufacturing a standardised support structure which can be reused throughout an entire building project, provided similar spans and loading requirements. The use of a curved surface onto which a false floor must be installed on also presents added difficulty in the assembly process. The development of a custom false floor system or integration of a flat top surface onto the shell itself may help to address this. As such, the problems present with funicular construction and its adoption in industry practice (that being increased labour costs compared to precast funicular construction) largely remains. However, there are avenues and options which needs to be further explored which may address these challenges. Nevertheless, the segmented fan concrete shell presents a potential means of enabling a lightweight and carbon-efficient flooring system that is compatible with component reuse and circular economy principles.

## Figures and Tables

**Fig. 19:**
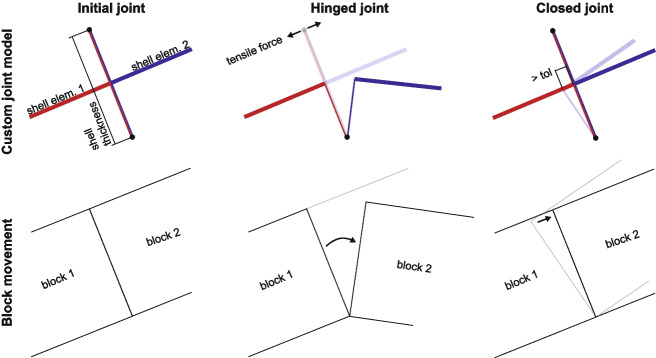
Schematic of custom joint behaviour which allows opening and closing of hinges

**Fig. 20:**
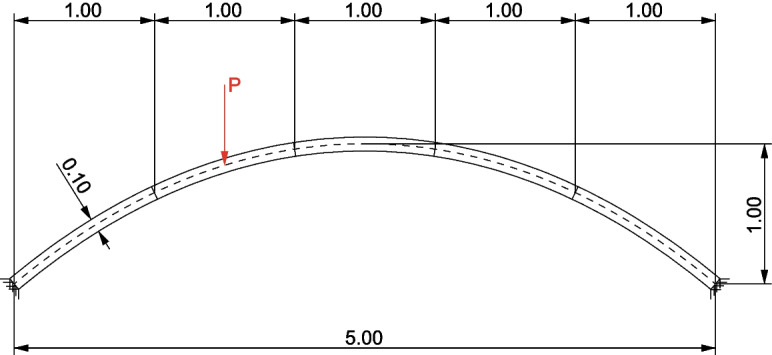
5-segment catenary arch used to validate the custom joint model

**Fig. 21:**
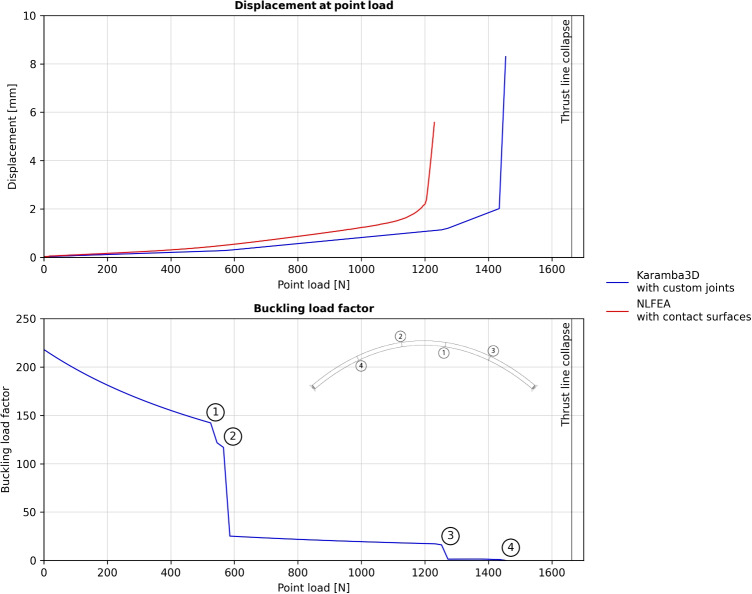
Comparison between results obtained from the custom joint model, an NLFEA, and thrust line collapse for the 5-segment catenary arch

## Data Availability

Additional data related to this publication are available at the University of Cambridge data repository at the following link doi.org/10.17863/CAM.96375.

## Appendix A Form-finding analysis method

A novel custom joint interface modelling approach was developed to explicitly capture the behaviour of the joints, inspired by similar interface modelling approaches used for rigid block analysis [53]. The behaviour of the joint is illustrated in Fig. 19 and is implemented in Grasshopper3D [25] relying on Karamba3D [24] as the solver. The interfaces are modelled using stiff beam elements connected at the extrados and intrados locations by nodes. The choice to use stiff beam elements is due to the lack of rigid beam elements in Karamba3D. In the presence of tensile forces at the ends of these beam connections, the joint is allowed to open, thereby allowing hinges to form. If an open joint is detected to displace such that the joint will close (i.e., the shells start to penetrate each other), the joint is then closed by reconnecting the beam connections. Using this approach, the joint is able to hinge at either end, close when needed, and also fully detach (if tension is registered at the beam connection at an already hinged joint). This nonlinearity is dealt with using an iterative and incremental analysis method based on the linear elastic analysis solver within Karamba3D. The loads are incrementally applied and at each load stage, the hinge states are modified until they are converged (i.e., no tensile forces are transmitted across interface nodes and no penetrations exist which suggests that the hinges should be closed). Unlike a fully non-linear analysis, the initial geometry and stress state are not modified at each load stage, merely the hinge and interface states. In addition, slipping between segments is not captured, only hinging and fully opening behaviour. Nevertheless, the devised analysis method provides a balance between a fast and simple linear elastic analysis and a fully non-linear analysis using contact surfaces which makes it compatible with population-based optimization algorithms and approaches (which was used as a form-finding strategy in Section “Shell form-finding and optimisation”).

The modelling approach was validated by modelling a 5-segment catenary arch with a cross-section of 100 mm by 100 mm spanning 5 m with a depth of 1 m. The material is taken to be similar to conventional concrete with a density of 2400 kg/m$^3$, a Young’s modulus of 30 GPa, and a Poisson ratio of 0.15. A downward point load is applied on the centroid of the second segment on the left until failure. While this structure is essentially a 2D problem, it was modelled in 3D with several shell elements spanning the out-of-place direction’s length. This was because the analysis method is targeted towards 3D structures and shells as opposed to arches. The geometry and loading of this arch are shown in Fig. 20.

The results of the analysis are shown in Fig. 21 (blue) alongside results from a nonlinear finite element analysis (NLFEA) using contact surfaces (red) [32] and the collapse load obtained from thrust line analysis (black). It can be seen that the custom joint model provides a collapse load in between the values obtained from NLFEA and the thrust line collapse analysis. This is reasonable as the NLFEA is able to account for the geometrical non-linearities that arise as the load increases, leading to a lower stiffness as the load increases and an overall lower collapse load. Conversely, the thrust line analysis assumes completely rigid segments. In this case, where the segments are quite large and slender relative to conventional masonry bricks or voussoirs, this assumption results in an overestimation of the collapse load. The custom joints used in the Karamba3D analysis provide a balance between these two analysis methods by allowing hinges to form and, as such, capture some of the non-linearities of the structure without having to perform a fully non-linear analysis.

Looking at the buckling load factor as the load is increased shows that the analysis method is also capable of predicting the loads and location at which hinges will form; once a hinge forms, the buckling load factor drops significantly. Capturing the drop in buckling load factor from the presence of hinges presents another advantage of the modelling approach compared to a fully connected linear elastic analysis which would yield unconservative results for a segmented structure.

## Appendix B 3D scans

3D scans were taken of the shell each time it was fully assembled. Using this data, the thickness distribution of the shell can be obtained and compared to the designed geometry’s intended thicknesses. The results of this are shown in Fig. 22. As MN1 and MN1M were constructed from the same set of conoid segments, the thickness distribution is almost indistinguishable. Overall, the thicknesses of the conoids are greater than intended. This is a common trend for segments sprayed using the ARCS fabrication method [13]. The thickness of the first conoid sprayed for MN1 and MN1M located in the bottom left can also be seen as higher compared to the rest of the conoids as they were fabricated prior to the 75% thickness correction factor being applied. The deviations compared to the intended thickness (normalised by the maximum thickness of 50 mm) are also shown in Fig. 22. Visible here is the characteristic grid caused by the sagging of the fabric formwork on the actuated pin-bed’s grid of carbon fibre strips. The variations in thickness between the different conoids of a shell (despite having the same robotic spray path trajectory) are due to the variations caused by the calibration of the machine settings which are performed manually for each separate spray session.
